# Moving window growth—A method to characterize the dynamic growth of crops in the context of bird abundance dynamics with the example of Skylark (*Alauda arvensis*)

**DOI:** 10.1002/ece3.4398

**Published:** 2018-08-11

**Authors:** Jörg Hoffmann, Udo Wittchen, Gert Berger, Ulrich Stachow

**Affiliations:** ^1^ Julius Kühn‐Institut Federal Research Centre for Cultivated Plants JKI Institute for Strategies and Technology Assessment Kleinmachnow Germany; ^2^ Leibniz Centre for Agricultural Landscape Research ZALF Müncheberg Germany

**Keywords:** crops, dynamic habitat quality, moving window abundance, moving window growth, Skylark, vegetation structures

## Abstract

Agricultural field crops differ in their vegetation height, coverage, and temporal development, affecting the abundances of bird species, which are often used as bioindicators. Although this relationship has been observed, no significant methodology exists to describe the dynamics of field crop growth on a landscape scale in connection with the abundance of indicator bird species that allows meaningful interpretation of bird abundance data with respect to crop vegetation parameters during the breeding season. In a field observation program, we monitored 2,900 ha of agricultural landscape to represent both the crop growth processes and the bird abundances. We measured these two parameters in the study area, dominated by winter wheat, winter rapeseed, maize, and fallow fields, and adapted the moving window approach to a new method of “moving window growth” to describe the dynamic development of height and coverage of the crops over time. Simultaneously, Skylarks (*Alauda arvensis*) territorial behavior was measured concurrently on the same fields and crops. Their dynamic abundance was documented over the breeding season. To test the relationship between crop growth and development and bird abundance, we applied a generalized linear model (GLM) in two ways: (a) without differentiation of crop species and (b) with differentiation of crop species. We found significant relationships between bird abundance and vegetation height and coverage with respect to both individual parameters and their interactions, even without differentiation of the agricultural crops. In general, increasing vegetation height and coverage, especially the interaction, led to decreasing bird abundance values. The model quality increased significantly by including differentiation of specific crops as an explanatory variable indicating a non‐homogenous situation between crops. Separate models for individual crop species revealed larger differences in model quality with best and least goodness of fit values for fallow fields and winter rapeseed, respectively. Because of the clear interactions between bird abundance, type of field crop, and vegetation height and coverage, it follows that both habitat suitability assessments of arable fields and the definition of favorable vegetation structures for farmland birds should be crop species‐specific.

## 1 INTRODUCTION

Since the beginning of systematic farmland bird monitoring in agricultural areas of rural European landscapes in 1980, a strong decline in bird populations has been reported (BMUB, 2015; Inger et al., 2014; Pe'er et al., 2014; PECBMS, 2009; Sudfeldt et al., 2013). Several drivers have been identified, including the intensive use of agricultural land (EU, 2007), especially consequences of long‐term pesticide use (Bright, Morris, & Winspear, 2008; Jahn, Hoetker, Oppermann, Bleil, & Vele, 2014; Taylor, Maxwell, & Boik, 2006), increasing density and higher yielding crops (Aebischer, Green, & Evans, 2000; Dicks et al., 2011; Sanderson, Kucharz, Jobda, & Donald, 2013; Wilson, Whittingham, & Bradbury, 2005), and decreasing crop diversity and increasing homogeneity of agricultural land, including the removal of unproductive, semi‐natural habitats (Benton, Vickery, & Wilson, 2003; Morelli, 2013).

Progress in plant breeding and agricultural management has led to high‐yield crops in arable areas; however, individual crop species often differ significantly in phenology and growth patterns. In Central Europe, the main crop growth period in spring and summer coincides with the time of territorial occupation and reproduction by farmland birds. Plant growth leads to continuous changes in the vegetation structure of crops, influencing the suitability of crop fields as bird breeding habitats, as shown by Weibel (1999), Weibel, Jenny, Zbinden, and Edwards (2001), and Schön (2011) with regard to nesting and feeding. Height and coverage are important characteristics of vegetation structure, and temporal crop development can be described with these parameters (Toepfer & Stubbe, 2001).

If bird abundance values can be related to specific vegetation structures, the suitability of crops as habitat for farmland bird species can be inferred. For example, Jenny (1990) found that vegetation coverage of over 50% strongly limits the ability of Skylarks to move on the ground as well as to fly into the vegetation. Similarly, Toepfer and Stubbe (2001) acknowledge the influence of vegetation height and coverage, especially temporal development, on bird abundance. Typically, Skylarks emigrate from habitats if the vegetation becomes too high and too dense, and the birds then switch to areas with less dense vegetation (Stöckli, Jenny, & Spaar, 2006). Therefore, some empirical approaches to enhance the habitat quality of crop fields aim at less high and dense plant coverage, e.g., in some parts of the crop fields (Dicks et al., 2011; Donald & Morris, 2005; Fischer, Jenny, & Jenni, 2007; Morris, Holland, Smith, & Jones, 2004; Stöckli et al., 2006).

In order to gather the appropriate information, empirical data on both vegetation structure and bird abundance are needed, taken simultaneously on identical arable fields.

There are many models used to describe crop growth (Asseng et al., 2013; Mirschel & Wenkel, 2007; Nendel et al., 2011; Poluektov & Terlev, 2007; Wenkel & Mirschel, 1991); however, most crop growth models focus only on anthesis, maturity, and especially on crop yield. They do not model the habitat structures characteristics like height and coverage of the vegetation. On the other hand, many bird monitoring programs in agricultural landscapes do exist without a parallel documentation or measurements of crop vegetation characteristics. As a consequence, the relations between crop growth dynamics and abundance dynamics of farmland birds are unclear, apart from some observations on very local and short time bases.

To solve this problem, a method is needed which encompasses (a) a data acquisition scheme, simple enough to be applied on a larger scale, i.e., on an appropriate number of fields; and (b) a crop growth model which characterizes the structural parameters of maize, winter rapeseed, etc., and allows to relate these to bird abundance data. To this end, we propose a novel crop growth model approach, which describes the growth process of the vegetation structures of specific crop species with a high temporal resolution. To assess the effects of crop vegetation parameters on the habitats of farmland birds, the time range of the breeding season should be covered.

**Figure 1:**
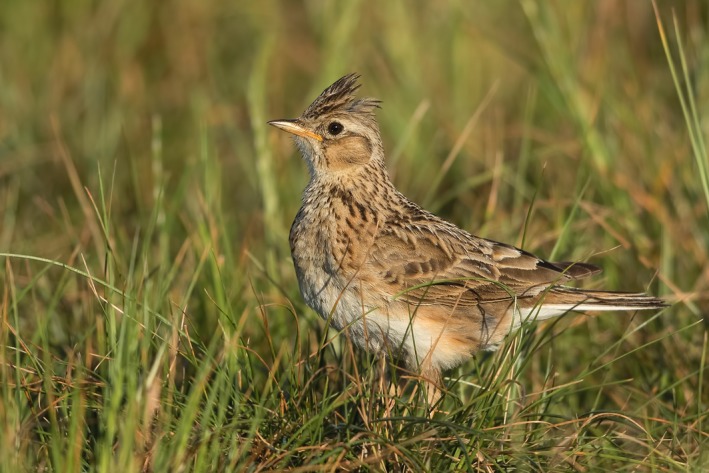
Skylark (*Alauda arvensis*) on a fallow field during the breeding period; Photograph: Steffen Fahl [Colour figure can be viewed at http://wileyonlinelibrary.com]

The received vegetation parameters (height, coverage) should be analyzed in relation to the simultaneously observed bird individuals with territorial behavior on the same fields. We take as an example the Skylark (*Alauda arvensis;* Figure 1), a typical farmland bird (BirdLife International, 2004; Gedeon et al., 2014), which is also an indicator species of the biodiversity of Central European farmland areas (Achtziger, Stickroth, & Zieschank, 2004; EBCC 2012). The relationships between crop vegetation parameters of various crop species and the observed Skylark abundances should reveal, by comparison, the similarities and dissimilarities of the vegetation structures of different crops during a period of Skylark abundance.

## 2 METHODS

### 2.1 Study area

The study area is located in Central Europe within the federal state of Brandenburg, Germany. The average annual temperature is 8.4°C, and the average annual precipitation is 520 mm. Fifty‐five percent of the total land area in Brandenburg is covered by agricultural landscapes, 69% of which is dominated by arable land, including 5%–7% semi‐natural small biotope structures. The main crops are winter cereals, winter rapeseed, and maize. Fallow fields, left idle for spontaneous vegetation development over several years, are found in varying proportions and have significantly decreased in recent decades (proportion of fallow fields in 1991 of 18.5%, 2007 of 11.75%, and 2010 of 4.15%; Anonymous, 2011). The study areas consisted of 29 plots of 1 km^2^ each (Hoffmann, Wittchen, Stachow, & Berger, 2016). The land cover types (crops and other habitats) are summarized in Table 1. Field maps were prepared based on the field geometries of all of the sampling plots using aerial photographs and field maps from the farms. The maps included the contours of all arable fields and small structures (biotopes), which were digitized and stored as polygon shapes (Hoffmann et al., 2016).

**Table 1:** Crops (number of fields (nf); ha) and other habitats (ha; ∑4.95%) found in the 29 plots (1 km² each)

Crops									
Winter wheat		Winter rapeseed		Maize		Fallow fields		Other crops	
nf	ha	nf	ha	nf	ha	nf	ha	nf	ha
24	609	25	689	21	649	28	337	38	415

Other habitats											
Woodland		Grassland		Small water bodies		Roads, field paths		Settlement areas		Bogs/swamps	
ha	%	ha	%	ha	%	ha	%	ha	%	ha	%
51.4	1.8	31.5	1.2	29.0	1.0	7.2	0.25	14.5	0.5	5.6	0.2

### 2.2 Investigating field crops and vegetation structures

Field surveys were conducted on a time interval (a, b) from March 16th to July 18th in 2010, concurrent with the mapping of Skylark individuals exhibiting territorial behavior (ITB; see Hoffmann et al., 2016). This four‐month period of plant growth was divided into eight sections of 15 days each, within which each plot was surveyed once. Each field survey was conducted by a trained biologist. The day on which an individual survey was conducted was chosen by the individual surveyor. Thus, the surveys of all plots were conducted on different days within the study time period of 15 days.

During each survey, on all plots the crops on a total of 143 fields, respectively, those parts of fields which were located inside the plots (19.3 ha average size, with a maximum of 96.8 ha) were documented, and two vegetation structural characteristics were determined: vegetation height (Vh) and vegetation coverage (Vc). Four classes of each of these parameters were distinguished (Table 2). The assignments of Vh and Vc in these classes were achieved by visual assessment while conducting line transects within the plots, with a distance of approximately 100 m between the transects. Small fields (10 fields <1 ha) were included by direct sampling, field by field. The spatial heterogeneity of the vegetation structures of the agricultural crops, e.g., due to variable soil conditions in the single fields or management effects such as tractor lanes, was recognized by estimating the share (%) of the field area that belonged to each of the Vh resp. Vc classes (see Tables 2 and 3). Data collection on one plot at one survey was thus linked to a transect survey length of approximately 8–10 km. The results were stored in databases (MS Access^™^).

**Table 2:** Classification scheme of vegetation structure: vegetation height (Vh) and vegetation coverage (Vc) as applied to each crop field for each survey

Vegetation structure parameters				
Variables	Class 1	Class 2	Class 3	Class 4
Vh (m)	0–0.25	>0.25–0.5	>0.5–0.75	>0.75
Vc (%)	0–25	>25–50	>50–75	>75

**Table 3:** Theoretical example to characterize the height of a crop in a study area (plot) on one date. Seven fields of this crop were found (columns 1–7), each with a specific distribution of areas belonging to the four height classes. For example, in field 1, 70% of the area had a crop height between 0.25 and 0.5 m (class 2) and 30% had heights between 0.5 and 0.75 m (class 3). The values in the columns add up to 100%. This was performed similarly for the vegetation coverage

Vegetation height Vh (m), classes 1–4		Field number within the plot						
		1	2	3	4	5	6	7
1	0–0.25 m	0	100	40	60	100	80	70
2	>0.25–0.5	70	0	50	30	0	10	30
3	>0.5–0.75	30	0	5	10	0	5	0
4	>0.75	0	0	5	0	0	5	0

### 2.3 Investigating bird data

The data surveys on Skylark were performed on the 29 1‐km² plots using mapping of bird individuals or pairs with territorial behavior (Hoffmann, Wittchen, Stachow, & Berger, 2013; Hoffmann et al., 2012, 2016). This method is based on the “territory mapping method” (Dornbusch, Grün, König, & Stephan, 1969; Fischer, Flade, & Schwarz, 2005; Oelke, 1968). All detected birds which did not exhibit territorial behavior, probably guests and resting birds, were excluded from the subsequent analyses. The field surveys for birds were conducted by the same person on the same days as the vegetation surveys, as described above. Then we applied the “moving window abundance” (Hoffmann et al., 2016) approach to the Skylark data for winter wheat, winter rapeseed, maize, and fallow fields.

### 2.4 Calculation of vegetation structures in the time course

**Figure 2:**
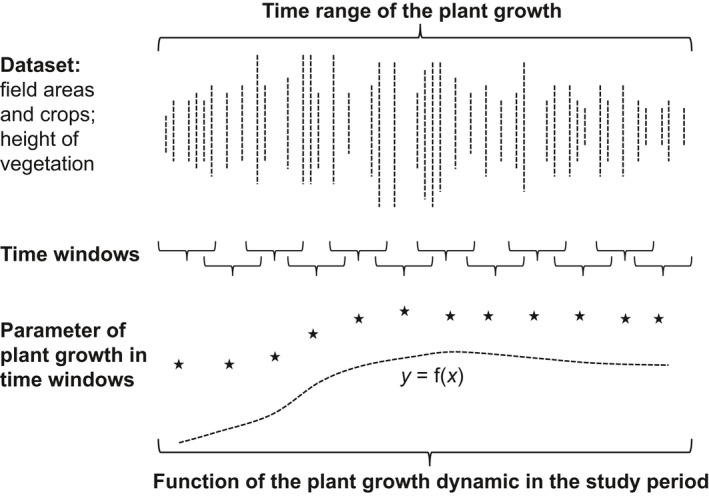
Scheme of the moving window growth (MWG), considering the time range of plant growth, datasets of plant growth parameters using the example of vegetation height, selected time windows, calculated parameters of plant growth within time windows, and the function of plant growth over the course of time. Figure modified for MWG after Hoffmann et al. (2016)

We modified the “moving window abundance” method (Hoffmann et al., 2016) to be applicable to plant growth, which we termed “moving window growth” (MWG). MWG characterizes the growth of study crops within a time interval (a, b) (Figure 2); in our case, the growth period was between March 16th and July 18th, which covers the breeding period of Skylarks. MWG was used to calculate daily values for the structural vegetation parameters Vh and Vc based on the field data (see above). It was necessary to compare the parameters to standardize the data for height (m) and coverage (%) to a dimensionless index between 0 and 1 for each parameter.

The time window in MWG was five days, and the average was assigned to the 3rd day. The shift of consecutive windows was one day, according to the specific overlapping moving window method (Hoffmann et al., 2016). The results are crop‐specific daily Vh – index (VIh) and Vc – index (VIc) values for the 121‐day time period from March 18th to July 16th. Finally, the functions that fit the values were calculated using SAS^™^ (NLIN procedure).

Based on this, the analyses were conducted in three steps, (a) to (c), as described below.

a) Course of plant growth for vegetation height and vegetation coverage

The course of plant growth assigns a numerical value, distinct for vegetation height Vh and vegetation coverage Vc, to each five‐day window based on the classification scheme explained in Tables 2 and 3. This is performed with respect to the area shares of each class. For example, if 75% of the area of a specific agriculture crop belongs to height class 1 (0–0.25 m), then the relative area of class 1 is 75 [rAh(1) = 75]. Combining all classes into vegetation indices (VIh for height, VIc for coverage) is accomplished using Equations (1) and (2).

VIh for height:

(1) $$VIh=0.01*\;\sum_{i=1}^{n}\frac{i-1}{n-1}*rAh\left(i\right)$$

[*n* = 4 = number of the vegetation height classes, see Table 2; *i* = individual height class (*i* = 1–4); rAh = relative proportion (% of the area) of height class I].

and

VIc for coverage:

(2) $$VIc=0.01*\;\sum_{j=1}^{n}\frac{j-1}{n-1}*rAc\left(j\right)$$

[*n* = 4 = number of the vegetation cover classes, see Table 2; *j* = individual cover class (*j* = 1–4); rAc = relative proportion (% of the area) of cover class *j*].

The indices are standardized so that all of the values are between 0 and 1. If all of the vegetation belonged to class 1, then the vegetation index would be 0; if all of the vegetation belonged to class 4, then the vegetation index would be 1 (see Table 2). All combinations of classes resulted in values between these numbers. Based on 121 values for time intervals (a, b), the functions of MWG for the vegetation indices height (VIh) and coverage (VIc) were calculated using SAS, with the NLIN procedure.

b) Impact of vegetation structure on Skylark abundance

Because individuals with territorial behavior (ITB) of Skylark and crop vegetation structural parameters were monitored simultaneously, the abundance values, expressed as the moving window abundance (MWA) (Hoffmann et al., 2016), could be directly connected to the vegetation structure, expressed as MWG for VIh and VIc. For both datasets, MWA and MWG daily values were available for the entire 121‐day time interval. A total of 5,539 ITB were recorded, mapped, and included in the analyses. The ITB was distributed to the crops as follows: winter wheat (WW), 1,507; winter rapeseed (WR), 1,220; maize (MA), 1,417; and fallow fields (FF), 1,395 (Hoffmann et al., 2016).

To analyze the effects of agricultural crops and their vegetation structures on the MWA of Skylark, we first determined the species main period during which the Skylarks were present in the area by analyzing fallow fields (FF) over time, because we concluded that this type of land use best represents the natural habitat demands of the Skylark (Hoffmann et al., 2012, 2013). Based on defined abundance classes and the calculated MWA of the Skylark [described by the function: *y* = −0.00014834*x*² + 0.10383*x* − 1.33087; *p *= 0.0002; *y* = value for the MWA; *x* = number of the day; *SD* = 1.56; abundance mean 4.6 and medium 4.55 ITB per 10 ha (Hoffmann et al., 2016)], we selected the time interval for the analysis period that resulted in the highest ranked habitat quality class (1) (see Table 4). This value indicates abundances of more than 4.25 ITB per 10 ha. In FF, abundances above 4.25 occurred for 99 consecutive days, between day 77 (March 18th) and day 175 (June 24th). Before day 77, we assumed immigration processes were occurring, and after day 175, we assumed emigration of Skylarks. Hence, the time period between these days represents the peak of Skylark presence within the agricultural landscape under study, and we thus compared the Skylark abundances of various agricultural crops within that time span.

**Table 4:** Abundance of Skylark individuals with territorial behavior (ITB) per 10 ha with associated habitat quality class (Hq) after Hoffmann et al., 2013

Skylark	Abundance (ITB/10 ha)				
	>4.25	>3.00–4.25	>1.75–3.00	>0.50–1.75	≤0.50
Habitat quality class (Hq)	1 – very high	2 – high	3 – intermediate	4 – low	5 – very low

We analyzed the effects of the explanatory variables VIh and VIc and their interactions on the MWA of Skylarks by applying a generalized linear model (GLM). The GLM was based on a Poisson distribution and log‐link function with Skylark abundance as a dependent variable and the log of the size of the investigation area as an offset variable.

First, we analyzed the effect of the vegetation parameters, including interactions on Skylark MWA for all investigated fields, without differentiation between the various agricultural crops. Next, we incorporated specific information about the various agricultural crops of WR, WW, MA, and FF as additional explanatory variables into the GLM. Additionally, we performed GLM to analyze the crops separately with respect to the two vegetation parameters. All of the calculations were performed using the statistics packages SPSS^™^, version 19. For GLM calculations, we documented the goodness of fit by deviance ratio, AIC (Akaike, 1973), BIC, and the *p* value for the considered interactions. We used AIC/BIC to select the appropriate models; the best model would exhibit the lowest AIC/BIC, indicating the best fit to the data. Parameter estimates were documented.

To identify crop‐specific vegetation characteristics, we compared Skylark abundances in the various crops when vegetation height was similar. This occurred in VIh between 0.05 and 0.20 (which corresponds to a height of 30–40 cm) and was realized during crop‐specific time periods, which we compared. Therefore, the crops provided a statistically homogeneous group. We tested group differences of VIh and VIc for WW, WR, MA, and FF and Skylark abundances by running non‐parametric Mann–Whitney tests for pairwise comparisons.

c) Habitat quality of field crops in the course of the Skylark breeding season

Based on the abundance classes defined according to Table 4 and the functions of MWA and MWG, the dynamics of habitat quality changes for each field crop during the Skylark breeding season were described by the sequences and lengths (in days) of different habitat quality classes.

## 3 RESULTS

### 3.1 Vegetation structures of field crops during the growth course

With the MWG growth model for the time interval (a, b), we collected data on vegetation height and coverage for 95% of all possible days in wheat, winter rapeseed, and maize and 97% in fallow fields (Table 5). The table also shows that approximately 1/3 of the areas covered by the respective crops are represented by each of the daily data.

**Table 5:** Proportion of days (% of all days during the observation period) on which vegetation parameters (height and coverage) were observed and registered; FF: fallow fields, MA: maize, WR: winter rapeseed, WW: winter wheat. For example, monitoring dates for the vegetation structure of winter wheat and bird abundances in wheat fields are available for 95% of all single days of the whole observation period, the average observed area being 196,1 ha per day

Parameter	Crops			
	FF	MA	WR	WW
Proportion of days (%)	97	95	95	95
Average area of arable land (ha)	111.7	234.3	231.9	196.1
Standard deviation	56.10	102.88	117.64	83.77
Median (ha)	100.8	232.6	218.6	191.8
Minimum (ha)	3.6	59.6	6.3	5.6
Maximum (ha)	245.4	441.9	550.4	416.4

**Figure 3:**
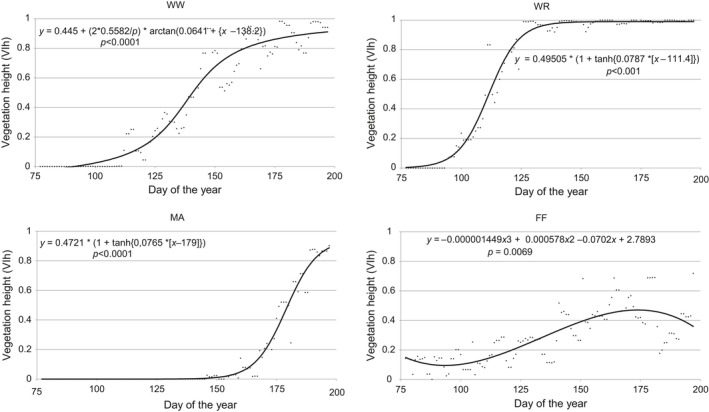
Moving window growth (MWG, dots) and function of growth at the example of height (VIh) within the time interval between day 77 and day 197 for winter wheat (WW), winter rapeseed (WR), maize (MA), and fallow fields (FF)

The growth curves of the agricultural crops differed clearly, as shown in Figure 3 for vegetation height (VIh as MWG) for WW, WR, MA, and FF. The functions of the MWG resulted in daily values for the whole monitoring period (77th to 197th day).

The function for WW follows an arctan curve, whereas WR and MA have a tangens hyperbolicus shape. In all field crops, a time period of little or no growth in height (VIh from 0 to approximately 0.1) is followed by a short phase of intensive growth, when VIh is >0.05 to approximately 0.8–0.9. However, the time periods of rapid growth differed between the crops (Figure 3, Table 6).

**Table 6:** The number of days on which a certain growth (height, expressed as VIh values) was reached in WW, WR, MA, and FF within the mapping period from the 77th to 197th day of the year

Crops	Defined VIh values			
	0.1	0.2	0.8	0.9
WW	116	126	163	191
WR	98	103	121	127
MA	166	171	191	–
FF	92	123	–	–

After the maximum height was achieved by WW and WR, the VIh showed few changes, indicated by VIh > 0.9. Maize had not achieved its maximal growth according to the VIh by the conclusion of the field‐mapping period (July 18th). The earliest and most rapid changes in VIh were seen in WR. In contrast to the three crops, in the semi‐natural grasslands of FF, higher vegetation was already seen at the beginning of the mapping period in the middle of March, with VIh > 0.1. This can be attributed in part to grasses and herbs left over from the previous year. The VIh then decreased to a value just slightly above 0.1 by the beginning of April (94th day) and then rose continuously and reached 0.2 on the 123rd day. The maximum VIh was 0.48, which was much lower than in the three crops. Also contrasting with the crops, no period of rapid growth could be observed. Thereafter, the VIh of FF dropped slightly by the end of the mapping period, again in contrast to the crops.

**Figure 4:**
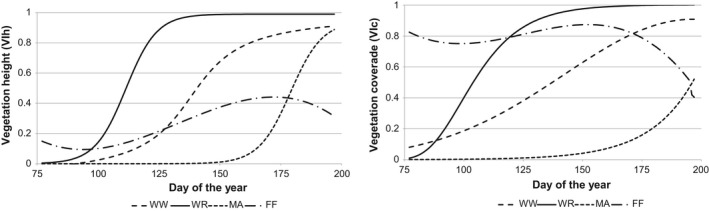
Dynamics of vegetation growth parameters, calculated by MWG, for winter wheat (WW), winter rapeseed (WR), maize (MA), and fallow fields (FF) within the time interval between the 77th and 197th day of the year. Left: function of the vegetation index “height” (VIh); right: function of the vegetation index “coverage” (VIc)

Similar to the height, the development of vegetation coverage also differed strikingly between the crops. Figure 4 shows the courses of MWG functions for the two vegetation structure indicators for the three crops and the fallow fields.

### 3.2 Vegetation structures as determining factors for Skylark abundances

The GLM showed significant main effects and interactions for vegetation height and coverage on Skylark abundance even when the crop species were not included (Table 7). The parameter estimates revealed negative regression coefficients for the VIh and VIc interaction.

**Table 7:** Results of GLM analysis: influence of vegetation parameters and their interaction on skylark abundance without the consideration of crop species

Statistic	Parameter	Value	Parameter estimation				
Goodness of fit	Deviance ratio	5.199	**Regression coefficient B**	**Standard error**	**95% Wald confidence interval**		**Sig.**
	AIC	3973.314					
	BIC	3988.990			**Lower**	**Upper**	
Omnibus test	Sig.(*p*)	0.000					
Test of model effects	Intercept (*p*)	0.000	−1.6	0.0135	−1.626	−1.573	0.000
	VIh (*p*)	0.000	1.089	0.0759	0.94	1.238	0.000
	VIc (*p*)	0.000	1.364	0.298	1.306	1.423	0.000
	VIh × VIc (*p*)	0.000	−2.92	0.0899	−3.097	−2.744	0.000

When the crop species (WW, WR, MA, and FF) were included as variables, the model fit was further enhanced (Table 8). Facing this higher model complexity the penalizing BIC value decreased and the deviance ratio was much lower and closer to 1, indicating a better goodness of fit.

**Table 8:** Results of GLM analysis: influence of crop species, vegetation parameters, and their interactions on skylark abundance

Statistic	Parameter	Value	Parameter estimation				
Goodness of fit	Deviance ratio	2.756	**Regression coefficient B**	**Standard error**	**95% Wald confidence interval**		**Sig.**
	AIC	3065.366					
	BIC	3128.068			**Lower**	**Upper**	
Omnibus test	Sig.(*p*)	0.000					
Test of model effects	Intercept (*p*)	0.000	−1.826	0.581	−1.94	−1.712	0.000
	Crop (*p*)	0.000					
	FF		1.663	0.203	1.265	2.060	0.000
	MA		0.271	0.061	0.152	0.390	0.000
	WR		0.324	0.072	0.182	0.466	0.000
	WW		0^a^				
	VIh (*p*)	0.000	−0.248	0.131	−0.504	0.009	0.059
	VIc (*p*)	0.002	2.472	0.199	2.082	2.862	0.000
	Crop*VIh (*p*)	0.000					
	FF*VIh		−2.610	0.725	−4.031	−1.188	0.000
	MA*VIh		2.750	0.424	1.919	3.582	0.000
	WR*VIh		5.359	0.290	4.791	5.927	0.000
	WW*VIh		0^a^				
	Crop*VIc (*p*)	0.000					
	FF*VIc		−3.130	0.312	−3.740	−2.519	0.000
	MA*VIc		−2.289	0.407	−3.087	−1.490	0.000
	WR*VIc		−2.913	0.256	−3.414	−2.412	0.000
	WW*VIc		0^a^				
	VIh*VIc (*p*)	0.000	−1.686	0.230	−2.137	−1.235	0.000
	FF*VIh*VIc		4.958	0.894	3.205	6.711	0.000
	MA*VIh*VIc		−11.209	2.270	−15.674	−6.743	0.000
	WR*VIh*VIc		−4.034	0.318	−4.658	−3.410	0.000
	WW*VIh*VIc		0^a^				
	Crop*VIh*VIc (*p*)	0.000					

a Set to zero, because parameter is redundant.

The separate GLMs of the four crops were significant but reveal large differences between model qualities (Table 9). Best model fit occurred for fallow fields, least for winter rapeseed. The winter wheat GLM quality was closer to fallow field, maize GLM performed at an intermediate quality. The regression coefficient for the VIh and VIc interaction for the three crops WR, MA, and WW showed negative values, whereas for FF positive values for this interaction occurred.

**Table 9:** Results of GLM analysis per crop: influence vegetation parameters and their interactions on skylark abundance

Crop	Statistic	Parameter	Value	Parameter estimation				
FF	Goodness of fit	Deviance ratio	1.560	**Regression coefficient B**	**Standard error**	**95% Wald confidence interval**		**Sig.**
		AIC	672.150					
		BIC	682.240			**Lower**	**Upper**	
	Omnibus test	Sig.(*p*)	0.000					
	Test of model effects	Intercept (*p*)						
	Test of model effects	Intercept (*p*)	0.402	−0.163	0.194	−0.544	0.218	0.402
		VIh (*p*)	0.000	−2.857	0.713	−0.426	−1.459	0.000
		VIc (*p*)	0.006	−0.657	0.240	−1.127	−0.188	0.006
		VIh × VIc (*p*)	0.000	3.272	0.864	1.578	4.966	0.000
Maize	Goodness of fit	Deviance ratio	2.804					
		AIC	789.320					
		BIC	799.496					
	Omnibus test	Sig.	0.000					
	Test of model effects	Intercept (*p*)		−1.555	0.178	−1.59	−1.520	0.000
		VIh (*p*)	0.000	2.503	0.403	1.712	3.293	0.000
		VIc (*p*)	0.605	0.184	0.355	−0.513	0.880	0.605
		VIh × VIc (*p*)	0.000	−12.895	2.267	−17.338	−8.452	0.000
WR	Goodness of fit	Deviance ratio	4.415					
		AIC	868.016					
		BIC	878.147					
	Omnibus test	Sig.	0.000					
	Test of model effects	Intercept (*p*)	0.000	−1.502	0.043	−1.586	−1.418	0.000
		VIh (*p*)	0.000	5.111	0.259	4.604	5.618	0.000
		VIc (*p*)	0.006	−0.441	0.160	−0.755	−0.127	0.006
		VIh × VIc (*p*)	0.000	−5.720	0.220	−6.151	−5.289	0.000
WW	Goodness of fit	Deviance ratio	2.231					
		AIC	735.875					
		BIC	746.006					
	Omnibus test	Sig.	0.000					
	Test of model effects	Intercept (*p*)	0.000	−1.826	0.058	−1.940	−1.712	0.000
		VIh (*p*)	0.059	−0.248	0.131	−0.504	0.009	0.059
		VIc (*p*)	0.000	2.472	0.199	2.082	2.862	0.000
		VIh × VIc (*p*)	0.000	−1.686	0.229	−2.137	−1.235	0.000

### 3.3 Vegetation coverage and Skylark abundance at similar vegetation heights for different field crops

To identify the impact of crop‐specific vegetation structures on Skylark abundance, we compared the vegetation coverage and Skylark abundance at a similar vegetation height (VIh) for the crops. This was the case for a VIh of 0.12–0.13 for the three crops and the fallow fields. The coverage (VIc) differed significantly between the crops, as did the Skylark abundances (Table 10), with contrasting groups of crops in each case; for abundances: group (a) FF and WW, and (b) MA and WR; for coverage: group (a) MA, (b) WR and WW, and (c) FF.

**Table 10:** Vegetation coverage and skylark abundance of different crops at similar height; a, b, c: crop groups after non‐parametric Mann–Whitney tests for pairwise comparisons of VIh, VIc, and abundance for crops, p < 0.05; (coverage: three groups; abundance: two groups); see 3.3

Crop	*n*	Index vegetation height VIh (ẋ)	Index vegetation coverage VIc (ẋ)	Abundance (ẋ)
Fallow fields (FF)	36	0.12	0.81^c^	4.91^a^
Maize (MA)	8	0.12	0.08^a^	2.44^b^
Winter rapeseed (WR)	10	0.13	0.42^b^	2.63^b^
Winter wheat (WW)	6	0.12	0.33^b^	3.53^a^

### 3.4 Abundance courses of Skylark in relation to the course of the vegetation structure development

**Figure 5:**
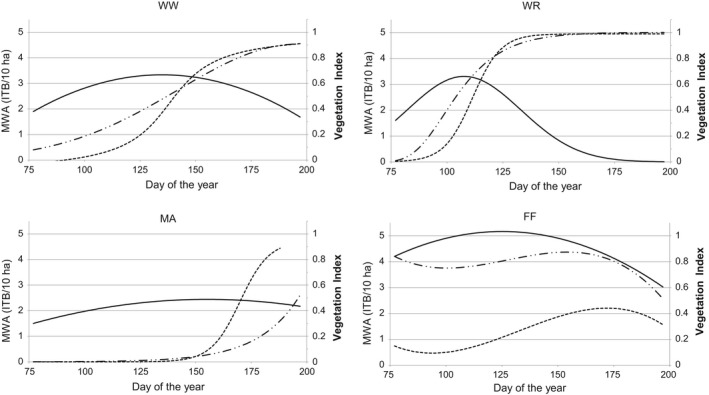
Moving window abundance (MWA: individuals with territorial behavior/10 ha) of Skylark (–) and moving window growth (MWG) (vegetation index of height VIh ‐ ‐.‐, vegetation index of coverage VIc ‐··‐·) of winter wheat (WW), winter rapeseed (WR), maize (MA), and fallow fields (FF)

The relationship between Skylark abundance and the development of crop vegetation structures, expressed as MWA resp. MWG, is shown in Figure 5. The parameters for vegetation structure were restricted to VIh and VIc.

The highest Skylark abundance values (maximum of the MWA function) were crop specific, with different parameter values for the vegetation structure as well as different dates (Table 11).

**Table 11:** Dates of maximum Skylark abundance per crop, i.e., individuals with territorial behavior/10 ha (WW—winter wheat, WR—winter rapeseed, MA—maize, FF—fallow field) and corresponding vegetation structures: VIh: indicator for plant height, VIc: indicator for coverage, see text

Crops	Day of the year	Maximum abundance	Vegetation structures	
			VIh	VIc
WR	108	3.3	0.37	0.60
FF	127	5.2	0.23	0.81
WW	135	3.4	0.37	0.48
MA	155	2.5	0.02	0.05

### 3.5 Comparison of the potential habitat quality of four agricultural crops during the Skylark breeding season

**Figure 6:**
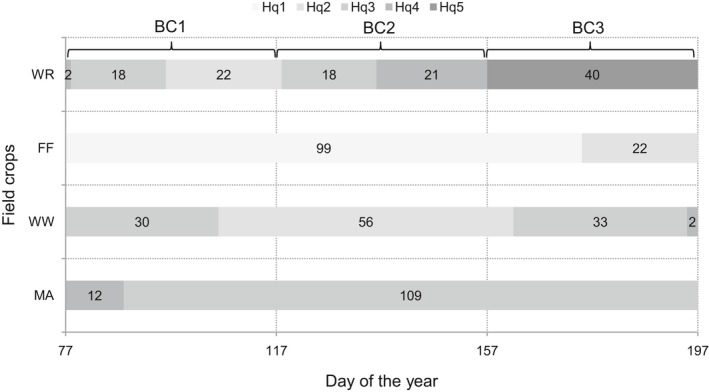
Time periods of potential habitat quality levels (Hq1 – very high, Hq2 – high, Hq3 – medium, Hq4 – low, Hq5 – very low) for Skylark in fallow fields (FF), maize (MA), winter rapeseed (WR), and winter wheat (WW); three successive breeding cycles (BC1, BC2, and BC3), each of 40 days, which are theoretically possible, are marked

Using the functional equation for MWA and MWG, the time periods for the five classes for the potential habitat quality (Hq1–Hq5, see Table 4) could be identified for each crop according to Skylark abundance (Figure 6). Moreover, three consecutive breeding cycles (BC1, BC2, and BC3; Hoffmann et al., 2016) of Skylarks, which are possible with an assumed duration of 40 days each, are marked in this figure. Different patterns of habitat suitability in the crops were obvious during the growth period (Figure 6).

Only FF exhibited the best potential habitat quality (very high; Hq1), which completely covered the first two breeding cycles (BC1 and BC2) and partially covered BC3. This potential habitat quality was already offered at the beginning of the breeding period on the 77th day of the year and ended after 99 days, on the 175th day. The second best class, potentially high habitat quality (Hq2), could be registered in the three crops at different times: in winter wheat, this occurred in the middle of the breeding period of 56 days; in winter rapeseed, this occurred in the second part of BC1, covering a very small part of BC2; and in fallow fields, this occurred at the end of the breeding period in the second part of BC3. In maize the majority of the time, only potentially medium habitat quality (Hq3) was registered, covering most of BC1 and completely covering BC2 and BC3.

## 4 DISCUSSION

### 4.1 Method to describe the growth patterns of agricultural crops

The new moving window growth (MWG) method, a modification of the MWA (moving window abundance) method (Hoffmann et al., 2016), may have the potential to closing the knowledge gap about interrelations between crop vegetation structures and farmland bird abundances in field crops. To this end, the synchronized collection of vegetation and bird data on identical fields seems to be of paramount importance.

With the MWG method, the daily values of the vegetation structures throughout the growth period were calculated and thus made usable to characterize the growth patterns. Height and coverage were thus expressed quantitatively for each day and thus for any time interval within the growth period, and thus allow for comparisons between different crop species.

The vegetation on fallow fields, which was not disturbed by any agricultural measure during the growth period, is composed of a variety of grass and herb species (Berger, Pfeffer, Kächele, Andreas, & Hoffmann, 2003; Hoffmann et al., 2012; Jüttersonke, Arlt, & Rischewski, 2008) and is a special case compared to the agricultural crops investigated in this study. These species‐rich and semi‐natural habitats do not exhibit a phase of “impulse growth” or other significant changes in vegetation structure within a short time period. The height, architecture, and phenology of the vegetation layer develop in a smoother and more heterogeneous manner due to species richness. This was observable throughout the whole monitoring period and sharply contrasted with agricultural crops, which aim for maximum yields. These conditions obviously led to very suitable habitat conditions for the Skylark on the fallow fields.

### 4.2 Interactions between vegetation structure and Skylark abundance

Our results support the view expressed by various authors (Jenny, 1990; Stöckli et al., 2006; Toepfer & Stubbe, 2001; Wilson et al., 2005) that vegetation height and coverage may serve as explanatory variables of Skylark abundance, independent of the specific crop species. These authors investigated the relationship between height and coverage as independent variables and inferred the general characteristics of the suitable vegetation structures of crops. A special distinction was made between autumn‐sown crops and spring‐sown crops because the former develop tall and dense vegetation cover much earlier in the year (Hiron, 2013). No author has made distinctions between crop species in respect of growth dynamics, height and coverage, and the development of respective growth functions to describe these processes. Toepfer and Stubbe (2001) do mention that interactions between vegetation height and coverage may exist with respect to Skylark habitat suitability, but they did not prove this. Our tests show a significant relationship between vegetation height and coverage as well as the interactions with Skylark abundance, even without reference to the crop species. This indicates the importance of these two characteristics for habitat suitability assessments. However, crop‐specific parameters, i.e., the inclusion of the crop species, significantly improved the statistical model.

Our analyses highlight the importance of the specific crop vegetation development for skylark abundance. Increase in interacting vegetation height and coverage in the cash crops (winter wheat, winter rapeseed, and maize) leads to decreasing bird abundance values. As crops may have similar height but differ largely in vegetation cover, resp. plant architecture, significant differences in habitat quality may result. Contrary to these crops increases in vegetation height and coverage in non‐productive fallow land may support higher bird abundances in general, with no decreases during the breeding period.

### 4.3 Habitat qualities of vegetation structures and Skylark abundance

The common method to characterize the suitability of a habitat for breeding birds is the determination of a single value for abundance, e.g., territories per 10 ha (Bauer, Fiedler, & Bezzel, 2005; Meichtry‐Stier, Jenny, Zellweger‐Fischer, & Birrer, 2014; and many others). These values may be valid for the whole breeding season only for certain types of fields in which the habitat conditions remain constant. However, this type of habitat quality assessment represents a static view, neglecting the dynamics within the season of both habitat structures and bird abundances (Hoffmann et al., 2016) as well as the interactions of the two. Some authors (e.g., Stöckli et al., 2006; Toepfer & Stubbe, 2001) have called for the inclusion of different phases of vegetation structural development and related bird abundances; however, an appropriate methodological approach has not been suggested.

With the example of Skylarks, we propose the classification of abundance values and subsequent projection with moving window abundance (MWA) and moving window growth (MWG) to allow for the delineation of time periods in which the potentially habitat suitability of the crop field is within defined limits. The temporal patterns of these suitability classes differ characteristically between and within crop species during the growth period (see Figure 6).

The moving window methods proposed here (MWG and MWA) are not based on a classification of crops (e.g., spring‐sown vs. autumn‐sown) but describe the development as a process that is individual for each crop species within a time interval (a, b). The application of the methods requires an appropriate sample size of both the study areas and bird individuals exhibiting territorial behavior to allow for statistical analysis of the differences between the growth dynamics of crops and bird abundances. The data and methods that do not accommodate these dynamics have limited explanatory value.

The results point to the importance of suitable vegetation structures and of crop diversity within a landscape for Skylarks. The diversity relies on a temporal aspect, i.e., the crop‐specific periods of high habitat suitability rotated between the crops within the breeding period, and a spatial aspect, i.e., respective crops are within the Skylark's flight distance. This also means that the theory that high crop diversity automatically leads to high abundance of Skylark (Daunicht, 1998; Engel, Huth, & Frank, 2012; EU, 2007; Tucker & Heath, 1994) can thus be attained only within limits set by the specific crops within the agricultural area (Chamberlain, Vickery, & Gough, 2000; Chamberlain, Wilson, Browne, & Vickery, 1999; Vepsäläinen, 2007). In areas with low crop diversity, temporally or spatially, land use types such as fallow fields, which have very high habitat suitability for Skylarks, obviously have the potential to partly buffer these deficits.

The temporal differences in maximum Skylark abundance values and the different potential habitat suitability classes identified in the crops clarify the appropriate time frame for optimized Skylark monitoring, in order to use bird species and their abundance dynamics as indicator for biodiversity components. In Germany, the suggested time period spans approximately 40 days, from the beginning of April to the beginning of May (Suedbeck et al., 2005). However, to adequately grasp crop‐specific habitat suitability dynamics, a longer monitoring period is necessary. As shown for winter rapeseed, high Skylark abundances (in our terminology: “Habitat suitability class 2”) can be found within a time interval of only 3 weeks. This indicator of high suitability, if taken alone, would suggest that winter rapeseed is a favorable crop for Skylarks. Taking into account the declining abundance in May and June, the overall assessment of this crop would be dramatically altered.

## 5 CONCLUSION

Of all of the factors that affect bird populations on agricultural landscapes, the crop species that are being grown and the spatial–temporal appearance of the respective vegetation structures are of significant importance. Therefore, methods that are applicable on the landscape scale are required to quantify and assess the habitat suitability of the crops and thus be able to better evaluate their functions for farmland birds and the potential to use data on bird abundances as ecological indicator for agricultural landscapes. Important questions to answer include, for example, what crops, with what vegetation structure, and during what time period would serve as suitable or less suitable habitat for birds? Furthermore, the importance of connectivity on the landscape, for example, via fields of the same crop species or spatial patterns of crop diversity with crops for farmland birds, could be analyzed more rigorously with these types of data. Answers to questions like these complement similar studies on the importance of marginal and unproductive habitats for bird species within agricultural landscapes (Morelli, 2013).

Because of the substantial effects of crop species on providing favorable conditions for Skylarks throughout the breeding season, we propose distinguishing crops by species‐specific characteristics of vegetation height and coverage. For meaningful assessments of whole landscapes, it is essential to consider the whole spectrum of important agricultural crops in that area; hence, we propose further systematic and empirical research. This should include identification of the effects of weather, climate, and soil conditions, as well as the management of agricultural land, including the application of pesticides and fertilizers onto the crop vegetation and hence habitat suitability for Skylarks and other bird species. It seems possible to integrate these approaches into ongoing monitoring systems, which could lead to enhanced efficiency and the explanatory power of the results.

Finally, the generation of the empirical data on crop vegetation should be performed and doable on a landscape scale, similar to typical bird monitoring schemes, so that data collections can be combined. In our view, this would significantly broaden the basis for fact‐based interpretation of bird abundance data, thereby creating appropriate hypotheses and experimental designs, and finally well‐based recommendations to support biological diversity in agricultural landscapes.

## CONFLICT OF INTEREST

None declared.

## AUTHOR CONTRIBUTION

J. H. developed the conception and design of the field study and drafted the article; U. W. and G. B. carried out statistical analyses of the data; U.S. contributed substantially to the interpretation of the data; all authors contributed substantially to the writing of the article and have read and approved the final version of the paper submitted.
